# Plant-Derived Natural Compounds as Bioactive Molecules with Beneficial Health Effects

**DOI:** 10.3390/ph19101606

**Published:** 2026-10-10

**Authors:** Diego Morales

**Affiliations:** Departmental Section of Galenic Pharmacy and Food Technology, Veterinary Faculty, Complutense University of Madrid, 28040 Madrid, Spain; diego.morales@ucm.es

The potential of natural compounds that can be extracted from a vast number of plant species in the field of human health has already been demonstrated in numerous studies [1,2]. Many plant components and metabolites have already been shown to contribute to reducing the risk of developing specific disorders and diseases, and even to help mitigate or alleviate symptoms once these conditions have developed [3,4]. However, the evidence supporting these effects is not always consistent, and the biological activity reported for plant-derived compounds does not necessarily translate into clinically relevant effects in humans. Factors such as compound concentration, chemical structure, bioaccessibility and bioavailability, metabolism, and the experimental model used may strongly influence the effects observed [5].

In this context, the strong correlation between natural compounds isolated from plants and health status has already been recognized by modern societies. Moreover, it is well known that the use of drugs may entail certain drawbacks, such as adverse reactions, side effects, and even a lack of specificity. In some cases, these drawbacks could potentially be overcome by the aforementioned plant-derived compounds [6,7]. Nevertheless, the assumption that natural compounds are inherently safer or more beneficial than synthetic drugs should be treated with caution, as plant-derived compounds may also present toxicological, pharmacokinetic, and bioavailability limitations, among others, as highlighted in the studies included in this Special Issue.

Thus, this field of research represents not only a major opportunity for scientific advancement but also a significant responsibility, as it needs to be addressed from multiple perspectives. On the one hand, progress must be made in the extraction techniques used to obtain these compounds from plant raw materials, including by-products. High extraction yields, together with efficiency and adequate selectivity, must be achieved while adhering to the principles of Green Chemistry through environmentally friendly techniques [8]. On the other hand, the fractions obtained must be characterized using technologies that enable the identification and quantification of the compounds present [9]. This step is particularly important because differences in extraction procedures and analytical methodologies can affect the composition of extracts and make comparisons between studies difficult. In addition, their biological activity and effects must be validated through in vitro and in vivo approaches—not only animal models but also clinical trials—and, whenever possible, accurate correlations should be established between individual molecules and their biological functions. In this regard, results obtained from in vitro and animal models should be interpreted cautiously, since the concentrations tested and the metabolic transformations occurring in vivo may differ substantially from those observed in humans. Likewise, the biological effects of complex plant extracts cannot always be attributed to a single compound, as interactions among different constituents may also contribute to the observed activity [10,11].

Within this framework, this Special Issue comprises six research articles and four reviews that take a cross-cutting and multidisciplinary perspective to address the different aspects outlined above. Taken together, these 10 contributions provide a comprehensive overview of the potential of plant-derived compounds in human health, while also underscoring the current weaknesses of the research in this field.

The diversity of the raw materials investigated by the contributing researchers also highlights the richness of the plant kingdom as a source of bioactive compounds [12]. Accordingly, leaves from species such as horseradish tree (*Moringa oleifera* Lam.), gum rockrose (*Cistus ladanifer* L.), mimosa (*Acacia dealbata* Link), and lemon verbena (*Aloysia citrodora* Paláu) have been investigated, together with the aerial parts of Irish fleabane (*Inula salicina* L.) and the fruits and flowers of black elder (*Sambucus nigra* L.).

These substrates were subjected to different technologies depending on the specific study, with the most noteworthy being conventional solid–liquid extractions using different solvents, solid-phase extraction, heat-assisted extraction, and ultrasound-assisted extraction. The target compounds investigated by the researchers were mainly phenolic in nature, including flavonoids, hydroxycinnamic and hydroxybenzoic acids, and curcumin and its derivatives, among others. In addition, Creteanu et al. (2024) conducted a literature review on the application of lignin as a biodegradable biopolymer in different formulation processes [13].

Regarding the models used to evaluate the biological activity of the compounds and extracts investigated, computational, in vitro and in vivo models can be highlighted. The latter involved experimental animals, including mice, rats and zebrafish. However, no results from clinical trials have been reported in this Special Issue, further highlighting the scarcity of human studies as a current limitation in this field.

Among the biological activities investigated, examples include the anti-inflammatory and antinociceptive effects of extracts obtained from the fruits and flowers of black elder in rats and mice, respectively [14], as well as the antioxidant and antimicrobial potential of extracts from the aerial parts of Irish fleabane, evaluated in vitro, including their ability to exert sun-protective properties and inhibit microbial biofilm formation [15]. Furthermore, Ciubotaru et al. (2024) investigated the behavioural and biochemical effects not only of curcumin but also of a mitochondria-targeted derivative, mitocurcumin, in a zebrafish model of epilepsy [16].

The remarkable results and insights reported in the 10 papers included in this Special Issue emphasize the current interest in these plant-derived compounds, as well as their promising future for applications in human health. At the same time, they identify current limitations in the field that should help shape the direction of future research. One potential area that deserves further attention is the utilization of plant by-products, which should be considered as valuable raw materials, enabling their revalorization while providing an important source of bioactive compounds [17]. Furthermore, the use of advanced and environmentally friendly technologies should be promoted. These approaches remain underexplored in this Special Issue but hold considerable potential, including through the use of supercritical fluids, microwave-assisted procedures, deep eutectic solvents, pulsed electric fields, and additional emerging technologies [18]. Moreover, the scope of research should be expanded to include other classes of plant bioactive compounds beyond (poly)phenols, such as peptides, lipids, alkaloids, and others. Finally, the promising results obtained in in vitro studies and animal models should be further validated through clinical trials to establish the actual effects of these natural compounds and fractions on human health.
